# Hair-Thread Tourniquet Syndrome: A Case Report and Literature Review

**DOI:** 10.1155/2012/171368

**Published:** 2012-10-15

**Authors:** Niroshan Sivathasan, Lavnya Vijayarajan

**Affiliations:** ^1^University College London Hospitals, London NW1 2BU, UK; ^2^LPMA (London Private Medical Associates), 130 Church Road, London SE19 2NT, UK; ^3^Watford General Hospital, Watford, Herfordshire WD18 9HB, UK

## Abstract

Though widely reported, Hair-thread Tourniquet Syndrome (HTTS) is poorly recognized. It refers to external, mechanical, circumferential constriction of an appendage, typically with an end-artery such as a digit, resulting in a “compartment syndrome-like” situation. HTTS is illustrated using the case of an infant. Children presenting with irritability should have their digits examined for signs of strangulation, with the awareness that numerous strands may be involved, with some buried in the skin. Early surgical-release must be performed if unwinding or simple cutting is unsuccessful.

## 1. Introduction

Hair-thread tourniquet syndrome (HTTS), coined in 1988 by Barton et al. [1], was recognized by at least the 17th century when a hair was reported as strangulating the glans penis. The Lancet officially published a case as long ago as 1832 [2], and HTTS has subsequently been well-established in medical literature [3]. Yet numerous clinicians remain unaware of it.

HTTS refers to hair (or textile-threads) getting firmly-bound around an appendage, typically with an end-artery such as a digit, causing a “compartment syndrome-like” situation and the possibility of ischaemic gangrene. There are a number of pseudonyms including tourniquet syndrome and toe tourniquet syndrome.

It is important to distinguish HTTS from congenital constriction band syndrome (amniotic band syndrome, Streeter's dysplasia), the latter being a rare congenital condition that is associated with other musculoskeletal disorders.

## 2. Case Report

An infant was referred by an emergency nurse practitioner (ENP) to the regional plastic surgery center for possible infection affecting the right middle finger. On presentation, the child was crying, and the digit in question was swollen and pale. The infant was a well-kempt white Caucasian and was interacting appropriately with the mother. The parents said that the child had become increasingly irritable since the day before and was now inconsolable. The mother had noted that the child was using the hand with the affected finger less, despite that hand being the more dominant one. There were no recent infections, no concerning past medical-history, and no known congenital issues, and the parents thought that blunt trauma was very unlikely. HTTS was considered, but a constricting thread was not obvious. A magnifying glass was not available, nor was depilatory cream. Using loupes (3x magnification) and a pen torch, the digit in question as well as the others was examined for HTTS. A glistening blonde strand was noted over the proximal phalanx, and subsequently hooked and cut free. Neurovascular observations were done overnight with discharge home the next day once the swelling had decreased, and after the digit was re-examined under magnification. Subsequent follow up was unremarkable.

## 3. Typical Characteristics of HTTS Presentation

Most cases of HTTS are presented to the emergency department or primary-care physician. Literature reviews have reported HTTS as affecting the following structures:

- digits (hand digits (24–47%) [4, 5], toes (25–43%) [4, 5]);
- external genitalia (penis (44%) [1, 6], Clitoris (6%) [3, 4, 7], labia [8]),
- head and neck (tongue, uvula, and neck [quoted from Saad et al. [6]]).

HTTS typically occurs in children, with one study quoting the mean age as 5.5 ± 4 months [9]; digital-wrapping is higher in those aged 4 days to 19 months [1], and penile-wrapping is higher in those aged 4 months to 6 years [1]. Excessive and protracted crying may be the only symptom in babies, although cases have been reported where infants have not shown irritability despite having HTTS for weeks [10]. Males and females are equally affected [11].

Cases have been reported in the literature involving ligatures tied to the penis for attempted control of enuresis [12]. Gypsies have been reported to wrap hair around digits to ward-off evil spirits [3]. 

## 4. Pathophysiology

There is lack of consensus as to exactly how, when unintentionally placed, the offending ligature wraps so tightly around a structure. Hair strands are much more likely to cause HTTS affecting the toes, whereas loose threads, such as from mittens, are usually responsible for the syndrome in hand digits. Although unknown why, the third toe is the most frequently affected digit [3]. Hairs are suppled and stretched easily when wet and contract when dry [13], and the circular configuration results in hydrogen-bonds giving a firmer hold. The high tensile strength of hair makes it an effective tourniquet. Should multiple digits be involved, then the doctor should have a heightened (though not absolute) concern for a deliberate aetiology [11].

The constricting band results in reduced venous and lymphatic drainage, causing swelling and oedema. Raised interstitial pressures may then reduce arterial supply, hence causing ischaemia and associated increasing pain and tenderness. The increasing swelling perpetuates this cycle in a positive-feedback fashion. There is a consequential risk of necrosis and autoamputation [14]. This process can occur over hours to weeks [15].

## 5. Associated Factors 

HTTS has been associated with postnatal telogen effluvium, which 90% of women experience at peak between 2 and 6 months after giving birth [10, 16]. Telogen is the phase in the hair cycle that involves hair shedding and is pronounced in the endocrine flux that ensues after childbirth. García-Mata and Hidalo-Ovejero (2009: 862) believe “that adhesion of hair and/or loose threads between digits is facilitated by home baths or by bathing in public swimming pools, due to the presence of hair remnants… [happening] much more frequently in the summer” [10].

Some authors have suggested that genital HTTS can be secondary to “gratification disorder” or “infantile masturbation,” that is, general self-exploration [7], especially considering that masturbation occurs in 90–94% of males and 50–60% of females [17]. 

## 6. Diagnosis

There is usually a delay of 3-4 days before the condition is recognized, and patients may delay presenting due to embarrassment [8] particularly when the genitals are involved. Discreet annular constriction frequently goes unnoticed and the skin is slowly penetrated, with neoepithelialization occurring over the offending item [10], thus making the diagnosis progressively more difficult [10, 15].

Differential diagnoses include infection, trauma, insect-bite, and allergic or irritant dermatitis. One should also consider the possibility of palmoplantar keratoderma and congenital constriction bands. Digital annular constriction affecting a toe, especially in one of African-descents, should raise the suspicion of the ill-understood condition called Ainhum (dactylolysis spontanea). 

## 7. Unusual Cases

Okeke (2008) reported a case of a 9-year-old boy with a 3-year history of penile HTTS, who presented not with strangulation but with florid granulation tissue and a tight urethral stricture. The patient had no “constrictive scar” and no discolouration or altered sensation distal to the granulation tissue over the HTTS, and this was postulated to be due to the ligature not being too tight [18].

Srinivasaiah et al. [19] described the case of a demented 80-year-old male who presented with a dusky and swollen inferior-third of a leg distal to a suspicious-looking circumferential zone with patchy areas of granulation. It was described as looking similar to lipodermatosclerosis, and examination of the circumferential area showed epithelialization over rubber bands. Thus it is important to remember that HTTS can be acute or chronic [20].

## 8. Immediate Management

Taking a casual approach to the release of the constriction-band can have devastating consequences, such as auto-amputation [21]. Having a high index of suspicion, particularly in children, is key to avoiding delays in treatment. Suggested management in the emergency department includes the following.

1. Pain control, and, if necessary, moderate sedation such as with ketamine.
2. Confidential clarification depending on the organ affected. For example, establishment of inappropriate sexual contact if the penis or clitoris is the affected structure. It is important to note that HTTS may easily be misinterpreted as abuse [22], when in fact most cases seem to be accidental in nature [10, 23].
3. Examination of the patient in a well-lit area. There are reports that circumferential erythema with a mild groove affecting digits has been misdiagnosed as infections, and appropriate treatment thus delayed [10]. Hussain (2008) described the case of a 6-year-old boy who had penile HTTS unsatisfactorily treated by a general practitioner over a period of two months, terminating in amputation for gangrene [24]. In the presence of uniform delineation, doctors are advised to examine the affected structure under a hand-held magnifying glass.
4. The constricting band may be mechanically removed, or depilatory agents may be used. If the constricting band is unclear or has involuted under the oedematous skin, then referral to the surgical team for examination under anaesthesia may be appropriate [8, 23].

If necessary, a short, longitudinal (paratendinous) incision placed perpendicularly to the constricting band [25] can be dorsally fashioned, paying heed to the anterolaterally-positioned neurovascular bundles. Antibiotic coverage should be provided as appropriate. Should formal surgery be required, then an additional, contralateral incision may be fashioned, and this should go all the way down to the bone to facilitate a definitive release, since severe inflammation can deeply bury the offending agent [10]. Post-decompression, the affected structure would be expected to heal well and without consequence, although flexion-deformity after ligature-release is a reported complication of prolonged digital HTTS [26].

If there are any concerns regarding the viability of the freed structure, then plentiful time for recovery should be allowed remembering that in infants in particular, occlusive vascular disease is rare and there is a propensity for tissues to recover.

### 8.1. Key Points for the Attending Doctor

- Children presenting with irritability should have their digits examined for signs of strangulation. Simple, but meticulous, unwinding may resolve the situation [27].
- Awareness that numerous strands of hair may be involved.
- Skin protection should be considered for excoriations and abrasions due to the tightly-wrapped hair-thread(s).
- Awareness that a secondary bacterial-superinfection may develop [10, 28].

## 9. Discussion

Whilst HTTS is uncommon (one center reported the annual incidence as 0.02% [9]), doctors must consider it in-order to facilitate prompt treatment to minimize the risk of amputation or functional loss. Doctors must exercise a lower threshold for concern in those with behavioural disorders, such as trichomania cognitive decline, such as Alzheimer's disease [29] and developmental disabilities, such as autism [4] in which communication may be limited.

Education is, once again, pivotal and must be targeted at:

1. parents, who should
  - be advised and encouraged to examine the insides of their child's clothing that covers the limbs, for loose threads or hair [10, 16]. Mothers should also be counselled about telogen effluvium and advised to check their infant's digits every few days or so [5],
  - avoid using coverings on their infant's extremities for extended periods of time without inspection [23],
  - launder children's clothes inside-out [11],
2. raising awareness amongst physicians in emergency medicine, general practice, and paediatrics, and surgeons in gynaecology, orthopaedics, and plastics.

In 1977, Douglas reported that chemical hair-removal agents may not be readily available in the medical environment [30]. Over three decades on, our experience suggests that some emergency departments still do not have the facilities to deal with such cases. One must remember that depilatory agents shall only work when the ligature is hair.

## 10. Summary

HTTS is neither new nor very rare, yet it remains poorly recognized. It is frequently a diagnostic dilemma [5], particularly when the strand's “excess” is broken off or cut thus causing reduced visible physical evidence of the ligature [14]. Accordingly, some authors advise that attempts at removing the offending agent in the emergency department may not totally relieve the strangulation and thus advocate the use of exploration under general anaesthesia [5]. If after magnified examination in a well-lit environment, there are no concerns of (subepithelial) constriction, then it would probably be prudent for the situation to be addressed in the emergency department, *with* subsequent monitoring over 24 hours under the care of a surgical team. This would avoid patients receiving unnecessary general anaesthetics. One must remember that if in doubt, it is safer to perform an examination-under-anaesthesia, be it local or general.

Strangulation should be considered when a swollen appendage is seen, particularly when there is a proximal circumferential depression. Take a careful and thorough history when a child presents under unusual circumstances, and consider child-abuse as a differential diagnosis [31]. The degree of damage is very variable and frequently correlates with the duration of strangulation by the mechanical-constrictor [24].
